# Host–Bacterial Interactions: Outcomes of Antimicrobial Peptide Applications

**DOI:** 10.3390/membranes12070715

**Published:** 2022-07-19

**Authors:** Asma Hussain Alkatheri, Polly Soo-Xi Yap, Aisha Abushelaibi, Kok-Song Lai, Wan-Hee Cheng, Swee-Hua Erin Lim

**Affiliations:** 1 Health Sciences Division, Abu Dhabi Women’s College, Higher Colleges of Technology, Abu Dhabi 41012, United Arab Emirates; h00413852@hct.ac.ae (A.H.A.); lkoksong@hct.ac.ae (K.-S.L.); 2 Jeffrey Cheah School of Medicine and Health Sciences, Monash University Malaysia, Jalan Lagoon Selatan, Bandar Sunway, Darul Ehsan 47500, Selangor, Malaysia; polly.yap@monash.edu; 3 Office of Campus Director, Abu Dhabi Colleges, Higher Colleges of Technology, Abu Dhabi 41012, United Arab Emirates; aabushelaibi@hct.ac.ae; 4 Faculty of Health and Life Sciences, INTI International University, Persiaran Perdana BBN, Nilai 71800, Negeri Sembilan, Malaysia; wanhee.cheng@newinti.edu.my

**Keywords:** bacterial membrane, secreting system, quorum sensing, antimicrobial peptides, antimicrobial resistance

## Abstract

The bacterial membrane is part of a secretion system which plays an integral role to secrete proteins responsible for cell viability and pathogenicity; pathogenic bacteria, for example, secrete virulence factors and other membrane-associated proteins to invade the host cells through various types of secretion systems (Type I to Type IX). The bacterial membrane can also mediate microbial communities’ communication through quorum sensing (QS), by secreting auto-stimulants to coordinate gene expression. QS plays an important role in regulating various physiological processes, including bacterial biofilm formation while providing increased virulence, subsequently leading to antimicrobial resistance. Multi-drug resistant (MDR) bacteria have emerged as a threat to global health, and various strategies targeting QS and biofilm formation have been explored by researchers worldwide. Since the bacterial secretion systems play such a crucial role in host–bacterial interactions, this review intends to outline current understanding of bacterial membrane systems, which may provide new insights for designing approaches aimed at antimicrobials discovery. Various mechanisms pertaining interaction of the bacterial membrane with host cells and antimicrobial agents will be highlighted, as well as the evolution of bacterial membranes in evasion of antimicrobial agents. Finally, the use of antimicrobial peptides (AMPs) as a cellular device for bacterial secretion systems will be discussed as emerging potential candidates for the treatment of multidrug resistance infections.

## 1. Introduction

The bacterial membrane is the protective barrier of the bacteria that helps transport proteins, virulence factors, solutes, and chemical signals [1]. There are various types of secretion systems in the bacterial cell that play important roles to either persist against host cells or to provide a response to the external environment. These secretion system attributes also enable the bacterial cell not only to compete with other microorganisms for host cell attachment, but also to provide immune evasion during the infection process [2].

Chemical signals are secreted through Quorum sensing QS in the bacterial membrane, which acts on cell-to-cell communication as an indication of population density [3,4]. Considering the important role played by the bacterial membrane in causing infections, use of antibiotics that target the bacterial membrane through different mechanisms would help in managing antimicrobial resistance [5,6,7].

Due to excessive use of existing antibiotics and the lack of new drug development, emergence of multidrug resistance (MDR) has caused a wide spread of infection worldwide, making MDR bacteria a threat to global health [8]. This mechanism of resistance may arise from antibiotic target modifications, such as the penicillin-binding proteins, and/or a change in the membrane permeability to antibiotics [9]. In addition, modification of the lipopolysaccharide charge can reduce the affinity of the antimicrobial to a membrane, causing resistance [10].

Recently, AMPS have shown promise against a large group of bacteria, as well as MDR bacteria, due to the AMPs’ ability to break down bacterial membrane using different models, which will be further discussed in Section 4 below [11].

## 2. Bacterial Membrane Interaction with Host Cells

Virulence factors are produced within the microorganism and secreted through membranes for adherence and for host cell invasion [12]. The process of bacterial attachment to host cells is the first step in initiating a series of biochemical reactions that include secretion of lethal substances and toxins from the bacterial cytoplasm and penetration of the host cell wall, resulting in infection establishment [13]. For example, virulence factors are important for the adhesion of *Escherichia coli* to the epithelium of the urinary tract, making it easier for *E. coli* to be transported through the urethra and uterus [14].

The host’s susceptibility to bacterial infection depends on the virulence of the bacteria’s virulence factors and the effectiveness of the host’s physiological and immunological systems. Infection begins when the virulence factors overcome the prevailing balance between them and the host’s immunity [12]. In addition, some bacteria possess properties that help them escape from the host’s immune system. Most of these processes are associated with the bacterial membrane [15]. Most bacteria use secretion systems which consist of transporters located in the bacterial membrane that are concerned with transporting proteins to the external environment or directly to the cells of the host. These proteins work to adapt the bacteria from their environment, create shelter, release DNA, and attack host cells [16]. Furthermore, a system called QS helps bacteria communicate with their external environment and coordinate their biological processes by secreting chemical signals through the bacterial membrane to compete or communicate with the bacteria in the surrounding environment [16,17]. This indicates that both the secretion and QS systems are closely associated to the bacterial membrane.

### 2.1. Secretion Systems in the Bacteria Membrane

Bacteria rely on secretion systems to secrete proteins to compete with neighboring microorganisms for ecological niches. For instance, the secretion system of *Pseudomonas aeruginosa* plays a role in triggering lung infections in cystic fibrosis patients, as the high viscosity of the mucus layer and disruption of mucociliary clearance are the main reasons why they are more susceptible to infections [18].

**Figure 1:**
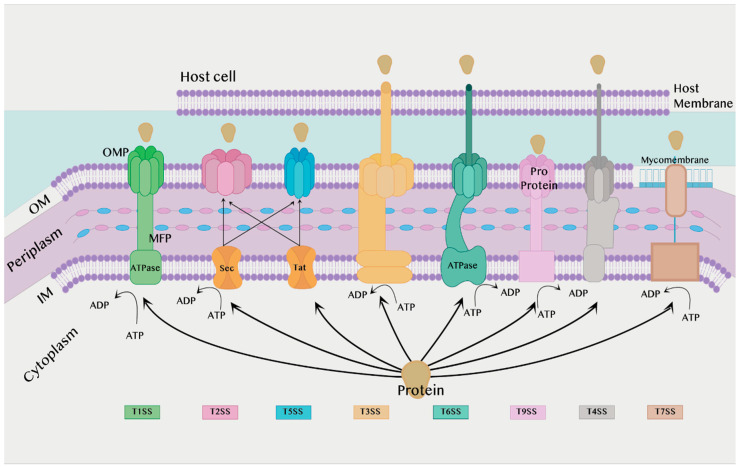
Different types of secretion systems. IM: Inner membrane. OM: Outer membrane. ATP: Adenosine triphosphate. ATPase: Adenosine triphosphatase. ADP: Adenosine diphosphate. MFP: Membrane fusion protein found in the T1SS. OMP: Outer membrane protein.

The secretion system is found in almost all bacterial species and is divided into eight types according to structure and activity: T1SS, T2SS, T3SS, T4SS, T5SS, T6SS, T7SS, and T9SS [16]. T8SS seem to have a limited role and, as it is not sufficiently understood, it will not be discussed in the current review. T1SS, T2SS, T3SS, T5SS, T6SS and T9SS are exclusively found in Gram-negative bacteria, while T4SS and T7SS can be found in both Gram-positive and Gram-negative bacteria, which will be discussed later. All secretion systems combined are shown in Figure 1 below.

#### 2.1.1. Secretion Systems in Gram-Negative Bacteria

Type I Secretion System (T1SS):

The T1SS is prevalent in Gram-negative bacteria such as *E. coli*, *Bordetella pertussis*, and *Vibrio cholerae*. This system depends on the secretion of proteins by interaction with only three components. Two of them are found in the inner membrane (IM), an adenosine triphosphate (ATP) binding cassette transporter (ABC) and the membrane fusion protein (MFP), while the third component is found in the outer membrane (OM), the outer membrane protein (OMP) [19,20].

Each of these three components plays a role in the substrate’s secretion process. ABC transporters catalyze ATP to provide energy for the secretion process, contribute to substrate recognition, and interact with membrane fusion proteins (MFPs) in IM [21]. The MFP extends over the periphery of the plasma and connects the ABC transporter in IM with the OMF in OM. It contributes to substrate selection [2]. The role of the OMP is to form a long channel through the OM to the external environment to support substrate release [22].

Hemolysin A (HlyA) T1SS is the first protein for which secretion was discovered in bacteria. The protein is found in strains of *E. coli* that cause gastrointestinal and urinary tract infections [19]. HlyA destroys host cells by facilitating the invasion process, subsequently causing holes in the cell wall, leading to destruction. HlyA is transported by the T1SS, which consists of three HlyA transporters, HlyB, HlyD, and Tolc. The HlyB protein belongs to the ABC transporters family, is located at IM, and serves to catalyze APT to provide energy, while Tolc is located at OM and serves to form a long channel through OM to secrete HlyA into host cells. The HlyD protein serves as a connecting channel between HlyB and Tolc [23].

Type II Secretion System (T2SS):

T2SS is found in many Gram-negative bacteria, and it secretes folded proteins from the periplasm to the OM. The type II secretion system is an important attribute for non-pathogenic human strains such as *Pseudomonas aeruginosa*, *E. coli*, *V. cholerae*, *Klebsiella species*, *Legionella pneumophila*, and *Yersinia enterocolitica*. Proteins are secreted to the inner membrane via the general secretory pathway (Sec) or the twin-arginine translocation pathway (Tat), whereby they are secreted from the periplasm to the OM via the T2SS [24]. In addition, the Sec system must fold the proteins during transport before they enter the T2SS [2].

The Sec pathway is responsible for the transport of unfolded proteins to the outer environment, periplasm, or IM. It consists of three components that work together in the secretion of proteins: the motor protein, protein targeting, and SecYEG translocase. Many different proteins are transported through this membrane, some of which increase the virulence of bacteria. In addition, there are many pathogens that rely on the Sec pathway to transfer virulence factors from the periplasm. Proteins that use the Sec pathway to be transported to the surrounding membrane or to the external environment contain a (SecB) specific signaling sequence, while proteins that remain in the IM contain a specific signaling sequence (SRP) [2,24].

In contrast to the Sec pathway, the Tat pathway neglects the transport of mainly folded proteins, and this pathway was first characterized in the early 1990s in chloroplasts in plants [25]. In this pathway, proteins cannot be secreted if they are not folded [2]. In Gram-positive bacteria, the Tat pathway is used to transport folded proteins directly to the external environment, while in Gram-negative bacteria folded proteins are transported to the periplasm of the plasm and then transported to the external environment via T2SS [16].

Type III Secretion System (T3SS):

Host cells have some physiological processes that help protect them from infection, such as maintenance of the cytoskeleton of the cell, which includes maintaining the cell structure and the phagocyte; however, pathogens that contain type III secretion systems can disrupt all these processes in order to cause infection [26]. They can transfer proteins in a single step by passing through the IM and OM to excrete the proteins [2].

The structure of T3SS is complex, containing several units of about 20 bacterial proteins. There are proteins that transfer other proteins to the cytoplasm of the host cell, which are considered virulence factors, and these proteins are called translocators, while the translocated proteins are called effectors [26]. Many pathogens use methods to evade trapping by the host’s immune system. It was discovered that the type 3 mechanism of the secretion systemT3SS has an effect which enhances the ability of bacteria to escape from phagocytes [27].

Type V Secretion System (T5SS):

The T5SS is considered peculiar because it is very small compared to other types and the energy source that supplies the secretion process is unknown, since no chemicals such as ATP are found in the periplasm. It also does not contain a stable ion gradient across the OM, which is why it is called an autotransporter (AT). T5SS is present in the OM of bacteria, so it requires the transport of proteins through the Sec pathway first, and then it is transported from the OM to the external environment by T5SS [28,29]. The structure of T5SSs are different based on features of their field organization, so T5SS/ATs have been divided into a number of subtypes from Va to Ve, and recently there may also be Vf [30,31].

Type VI Secretion System (T6SS):

Gram-negative bacteria possess several secretion systems that assist them in the invasion of host cells, one of which is the T6SS. T6SS is one of the complex systems that transports the effector to the external environment through the bacterial membrane. The effector secreted by T6SS not only attacks eukaryotic cells, but also targets other bacteria in order to compete for survival and cause infection and disease [32,33]. Effector delivery methods are categorized, whereby the effectors can be classified as specialized effectors or cargo effectors. The specialized effector binds with the C-terminus of T6SS structure proteins such as Hcp, VgrG, or PAAR for translocation into target cells. It is believed that specialized effectors that fuse with Hcp-VgrG-PAA-associated effectors are released in a single lethal shot into other bacterial or eukaryotic cells [32,34,35]. *Yersinia pseudotuberculosis* has been studied to show a different feature in T6SS-4, in that it is able to transport zinc (Zn^2+^), which plays an important role in immune system resistance and anti-stress, contributing to the survival of bacteria in harmful environments. Zn^2+^ transport depends on the classic ZnuABC transporter with T6SS-4 and yezP, and the results showed that any disruption of these important transport elements leads to the bacteria losing their virulence against mice [36].

Type IX Secretion System (T9SS):

T9SS is responsible for the secretion of effector proteins such as proteases, adhesins, cellulases, chitin, and surface layer proteins. T9SS is found in species of periodontal pathogens such as *Porphyromonas gingivalis* that use T9SS to secrete up to 30 effector proteins. T9SS is the primary determinant of virulence of oral pathogenic bacteria species relevant to acute periodontal disease in humans and animals [37,38]. *P. gingivalis* and *Tannerella forsythia* are the most common pathogens of periodontal disease and chronic periodontitis. In *T. forsythia*, it secretes a KLIKK proteases which is a proteolytic enzyme by the T9SS. KLIKK proteases participate in the invasion of the host’s integumentary system in order to protect the bacteria, while in *P. gingivalis*, T9SS is used to secrete gingipains, which are proteolytic enzymes [39].

#### 2.1.2. Secretion Systems in Both Gram-Negative Bacteria and Gram-Positive Bacteria

Type IV Secretion System (T4SS):

The T4SS is found in Gram-positive and Gram-negative bacteria, as well as in Archaea [40]. T4SS is characterized as being able to transport DNA along with proteins, whereby the DNA is transported without exposure to the outside of the cell. This system consists of two main components: the conjugation system and effector translocations. The conjugation system acts as a channel for the transfer of antibiotic resistance genes between bacteria, while effector translocations serve to transfer virulence factors to the host cell [16]. In Gram-negative bacteria, T4SS is divided into two types, T4SSA and T4SSB. In addition, Gram-negative and Gram-positive derived T4SSs were divided into eight classes based on detailed phylogenetic analysis [41]. It was discovered that the T4SS that is present in the Xanthomonadaceae family differs from the other T4SSs. The reason behind this is that the type IV secretion system present in *Xanthomonas citri* can secrete proteins that kill other Gram-negative bacteria in a contact-dependent manner [42]. T4SS was studied under a transmission electron microscope, where it was found that the basic molecular structure of the Dot/Icm secretion system type IV, consisting of at least five proteins encoded by DotC, DotD, DotF, DotG, and DotH, has a ring structure that is encoded by *Legionella pneumophila*, which is one of the intracellular opportunistic pathogens [43].

Type VII Secretion System (T7SS):

*Mycobacteria* and *Corynebacteria* species are resistant to the immune system and antibacterial drugs, and the reason for this is due to the presence of a thick waxy layer on the surface of the OM called the mycomembrane. There is a type VII bacterial secretion system which has effective mechanisms that help these bacteria secrete proteins through mycomembrane to the outside of the cell. The presence of the T7SS was first observed in *M. tuberculosis*. Recent research proved the presence of T7SS in non-mycomembrane bacteria such as *S. aureus*, Group B *Streptococcus*, *Bacillus anthracis*, and *L. monocytogenes*. The core T7SS structural components are encoded via gene clusters [2,44]. Type VII (T7SS) secretion systems in Group B *Streptococcus* contribute to the secretion of proteins that act as virulence factors, host toxicity, and killing of a number of bacteria in a few genera. Spencer and co-workers helped to understand the role of T7SS in the pathogenesis of GBS, as it is involved in a large part of the host’s cell interaction with Group B *Streptococcus*, as well as influencing the functions of the T7SS effector secreted by other Gram-positive bacteria [45]. Furthermore, the presence of T7SS in *Staphylococcus aureus* contributes to the resistance of antibacterial secreted by the host cells, as it has been shown that *S. aureus* lacking T7SS was killed by antimycotic fatty acids produced by the host cells [46].

### 2.2. Quorum Sensing (QS)

QS is a process of cell-to-cell communication through secretion of chemical signaling molecules that enable bacteria to recognize their surroundings [4]. QS helps bacteria regulate the population density of bacteria in their external environment by modifying their behavior through gene expression in response to chemical signals secreted by the bacteria. QS enables communication with other bacteria to live as multicellular organisms and collective interaction with host cells, and also helps meet the needs of given species for living in a particular niche [3,47,48]. The chemical signaling molecules called autoinducers are produced and released by bacteria, where different types of autoinducers are produced from different bacteria species as shown in Table 1. Bacteria then measure and respond to the accumulation of autoinducers in the environment. For example, when bacteria detect that the accumulation of a lower threshold concentration of these autoinducers has a stimulatory effect, they alter their gene expression to change their behavior accordingly [49].

Acute hepatopancreatic necrosis disease (AHPND), which is also called early mortality syndrome (EMS), appeared in Asia in 2009, which was the reason behind the destruction of the shrimp crop in East Asian countries and Mexico [50]. *Vibrio* spp. is the cause of AHPND, as it contains the pVA1 plasmid responsible for production of two toxins, PirAVP and PirBVP, that lead to symptoms of AHPND [51]. Bacteria precipitation may be affected by the environmental conditions around it. In the early log phase of the growth curve, the *Vibrio* strains showed an increase in the gene expression of PirAVP and PirBVP proteins, and this persisted into the log phase. It was discovered that the change in gene expression of PirAVP and PirBVP proteins was regulated by QS [52].

#### 2.2.1. QS in Gram-Negative Bacteria

QS is used by both Gram-negative bacteria and Gram-positive bacteria to communicate with each other and their environment, but there are differences in the mechanisms. First, we will focus on quorum sensing in Gram-negative bacteria, and then later on quorum sensing in Gram-positive bacteria. There are some common features of quorum sensing in Gram-negative bacteria.

In Gram-negative organisms, autostimulants (AIs) are synthesized from S-adenosylmethionine (SAM) as molecules such as acyl-homoserine lactones (AHLs), which are the most common class of AIs. They consist of an acyl-homoserine lactone ring and an acyl-carbon chain, the length of which affects the stability of the AHLs. LuxI family enzymes are the main producers of AHLs. They function as AHL synthases and catalyze the acylation of SAM by the acyl carrier protein (acyl-ACP). In this reaction, SAM provides the amino group and the acyl-ACP provides the acyl group for the synthesis of AHLs, which are released to the outside via the cell membrane and act as co-signaling molecules [47,53,54,55].

There are specific receptors in the cytoplasm and IM that associate with autoinducers; these receptors associated with AHLs aid gene expression in QS. For example, LuxR-type receptors in the cytoplasm are transcription factors. LuxR binds with AHLs to form a complex that binds to DNA to regulate bacterial gene expression. In *P. aeruginosa*, the LuxR/LuxI-type system contributes to communication with other cells [56,57].

Furthermore, receptors in the IM and cytoplasm regulate hundreds of gene expressions through QS. Receptors can regulate genes that directly influence the formation of virulence, biofilm, and the biological and physiological processes of bacteria. QS receptor molecules regulate gene expression through a mechanism called autoinduction. It is a mechanism that enhances gene expression in all bacterial populations by increasing the synthesis of autoinducers, resulting in an increased population at the end of the response [57]. 

#### 2.2.2. Quorum Sensing in Gram-Positive Bacteria

**Figure 2:**
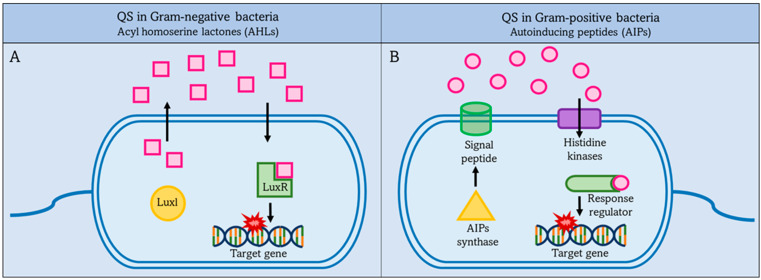
QS systems in Gram-negative and Gram-positive bacteria. Left figure (**A**): Gram-negative bacteria secrete acyl-homoserine lactones (AHLs) as autoinducers, which are synthesized by Luxl and then pass through the bacterial membrane into the external environment. Once the AHLs reach a threshold level, they activate intracellular LuxR to activate target gene expression. Right figure (**B**): Gram-positive bacteria secrete autoinduction peptides (AIPs), which are synthesized by AIPs synthase and then pass through the bacterial membrane into the external environment. Once the AIPs reach a threshold level. This is detected by a specific protein, histidine kinases, and activates the intracellular regulator. The response regulator leads to increased expression of the target gene.

QS in Gram-positive bacteria uses short peptides as autoinducers to determine population density as shown in Figure 2 [58]. These autoinducer peptides (AIP) are synthesized by the ribosome and then modified to be active. It is released into the external environment with the help of the ABC transporter located in the IM [59]. Autoinducers accumulate in the external environment until a threshold concentration is reached, and this is recognized by a specific protein, histidine kinase. Then gene expression is preceded in the bacterial population in response to the concentration of autoinducers. The higher the concentration of autoinducers in the external environment, the greater the bacterial population density [60].

There is a family of transcriptional regulators associated with peptides to help coordinate gene expression. This is the RRNPP family, which consists of Rap phosphatases, NprR sensor, PlcR, PrgX, and finally Rgg, which was added later. The RRNPP family is found in *Bacillus*, *Streptococcus*, and *Enterococcus* [60,61].

It was determined that there is a relationship between QS and the transformation of commensals into pathogens in *S. pneumoniae*. *Streptococci* species Rgg/SHP is widely spread among their species, as it was discovered that the *S. pneumoniae* D39 Rgg0939/SHP system regulates transcription of one gene group, and it was established that QS in *Streptococci* species is responsible for regulating exopolysaccharide synthesis from a distinct site [62].

#### 2.2.3. The Role of Quorum Sensing in Biofilm Formation

**Figure 3:**
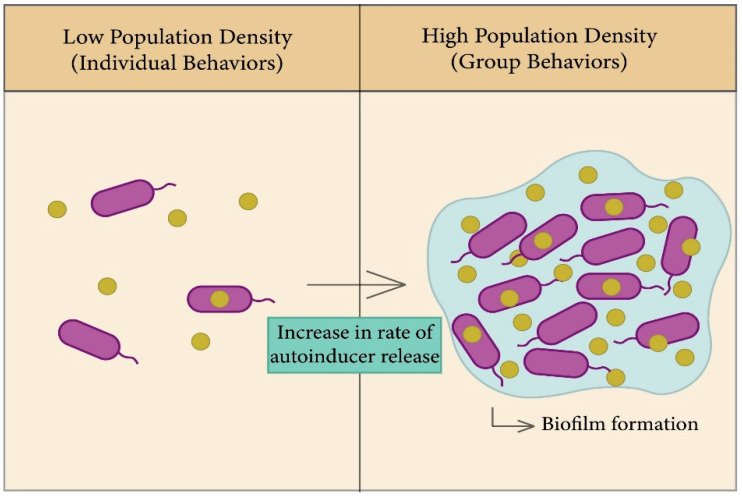
General principle of the QS system. **Left figure**: Each species begins to secrete autoinducers into the external environment, and the population density increases. **Right figure**: Increased rate of autoinducer release to form biofilm due to gene expression.

In bacteria, biofilms are one of the most important factors that lead to antibiotic resistance and a reason for persistence of infection [63]; the general principle of biofilm formation is illustrated in Figure 3. Biofilms consist of a group of the same or different types of bacteria that adhere to each other and to the surface, whereby they assemble together by polymeric matrices consisting of secreted proteins, sugars, lipids, and extracellular DNA [64]. QS helps in increasing the rate of biofilm formation, in addition to virulence factors. Bacteria can trap secreted autoinducers from the quorum sensor in biofilm gene expression in order to enhance bacterial survival in the colonizing environment [63,65,66].

Biofilms are formed through four steps: binding, cell-to-cell adhesion, maturation, and dispersal [63]. Once biofilms are formed, they are accompanied by their extirpation, as they have the ability to resist antimicrobials, as the formation of cell membranes is one of the mechanisms of bacterial adaptation to their environment. The study of *Streptococcus gordonii*, which demonstrated the role of QS systems in the formation of cell membranes, found a defective mutation in biofilms which have a transposon insertion in the comD gene, which is responsible for encoding the sensor protein (histidine kinase) in Gram-positive bacterial QS. As such, the formation of biofilm is highly dependent on communication between cells via QS [67,68].

**Table 1:** Autoinducers molecules from different bacteria.

Autoinducers	Microorganisms	Receptors	Phenotypes	References
Autoinducing peptides (AIPs)	Gram-positive bacteria	Response regulator	Genetic competence.	[69]
Autoinducer-2 (AI-2)	Many Gram-negative and Gram-positive bacteria	LuxP, LsrB, and dCACHE	Virulence, biofilm formation, and protease.	[70,71]
CAI-1 autoinducer	*Vibrio*	CqsS	Virulence, biofilm formation, and protease.	[71,72]
HAI-1	*Vibrio harveyi* and *Vibrio parahaemolyticus*	LuxN	Biofilm formation, bioluminescence, TTS, and protease.	[73]
Acyl homoserine lactones (AHLs)	Gram-negative bacteria and commensals	LuxR	Elastase, biofilm formation, virulence factors, and exotoxins.	[74,75]
Competence-stimulating peptides (CSPs)	*Streptococcus pneumoniae*	ComD	Biofilm formation, and virulence.	[76]
comX-inducing peptide (XIP)	*Streptococcus mutans*	ComR	Antibiotic tolerance, genetic competence, and dormancy.	[77,78]
ComX	*Bacillus subtilis*	ComP	Protease and biofilm formation.	[79,80]

## 3. Bacterial Membrane Interaction with Antimicrobials

There is an urgent need for the development of new antibiotics in order to effectively treat infections caused by MDR bacteria [81]. As depicted in Section 2, bacterial membranes play crucial roles in cell physiology and survival, thus justifying the rationale for choosing bacterial membrane as the drug target. Furthermore, the bacterial membrane differs significantly in structure from the mammalian membrane. This means antimicrobials will have greater selectivity for bacterial membranes [81,82].

Antimicrobials that target bacterial membranes are generally known to be lipophilic and actively react to the lipid bilayer of the bacterial membrane. Experts believe that the best treatment for slow-growing bacteria such as *Mycobacterium tuberculosis* is to design an antibiotic that targets the regulation of the bacterial membrane or the functions of “membrane-bound enzymes”. These antibiotics can also disrupt the synthesis of proteins in the bacterial membrane [5].

### Phospholipid Targeting by Antimicrobials

There are few antibacterials that target the bacterial membrane. The biggest challenge faced by the manufacture of antimicrobials that target the bacterial membrane is the selective toxicity of antibiotics, in which, ideally, they should be highly effective against the bacteria, but have low or no toxicity to humans [6].

Phospholipids are one of the types of lipids found in bacterial membranes. They are the main component of the IM in Gram-negative bacteria, as they play an important role in preventing antibiotics from entering the bacteria by creating a unique double membrane. Cardiolipin (CL), phosphatidylglycerol (PG), and phosphatidylethanolamine (PE) are the most prevalent classes of phospholipids in bacterial membranes, and this diversity contributes to the design of antibacterials that target specific classes of lipids in the membranes without attacking mammalian cells [81,83].

There are different ways to target phospholipids that result in changes in the properties of bacterial membranes, the most important of which are the bulk physical properties of the membrane. Firstly, clustering, for example, changes the assembly of fats, which may result in the poisoning and killing of bacteria; this may be the result of impeding the interaction of lipids with other components in the membrane, changing the distribution of lipids, or forming defects in the lipids cluster [81]. Secondly, the curvature of the bacterial membrane is important in determining the activity and interaction of various membranes; its property affects the antimicrobial function [84]. Richard M. Epand and co-workers discussed how membrane curvature can affect the properties of a membrane protein, and therefore investigated the relationship between protease activity and modulation of membrane curvature using nuclear magnetic resonance [84]. The synthetic peptide MSI-367 [(KFAKKFA) 3-NH2] was investigated by 2H NMR and 31P NMR. The preferential increase of the phospholipid head region led to an expansion of this region and thus to a positive curvature. Moreover, this was confirmed by differential scanning calorimetry (DSC) measurements, which revealed that the peptide inhibits conversion of the lamellar phase of phosphatidylethanolamine to the hexameric phase, indicating its ability to inhibit the formation of structures with negative curvature [84,85]. In addition, another feature of the peptides that led to a change in membrane curvature that was observed was the discrepancy between the thickness of the bilayer and the length of the peptide. It was partly demonstrated by 31P NMR that the model WALP peptide can enhance negative curvature [84,86].

Lipophosphonoxins (LPPOs) are synthetic compounds designed to target the cytoplasmic membrane. There are two generations of LPPOs; the first generation of LPPOs (LPPO I), which show activity against Gram-positive bacteria but have no effect on Gram-negative bacteria; and the second generation of LPPOs (LPPO II), which are active against Gram-negative bacteria. Both generations of the LPPOs are able to form pores that permeabilise the bacterial membrane, but LPPO I is not capable of bypassing the OM with LPS, thus it is insensitive against Gram-negative bacteria [87]. This highlights the importance of OM and LPS as primary hurdles to overcome before the antimicrobial compounds gain access to their cytoplasmic targets. Moreover, compound interaction with OM and LPS, as well as the resultant membrane potential, should be taken into account when designing new antimicrobials.

## 4. AMPs as Molecules Targeting Bacteria

**Figure 4:**
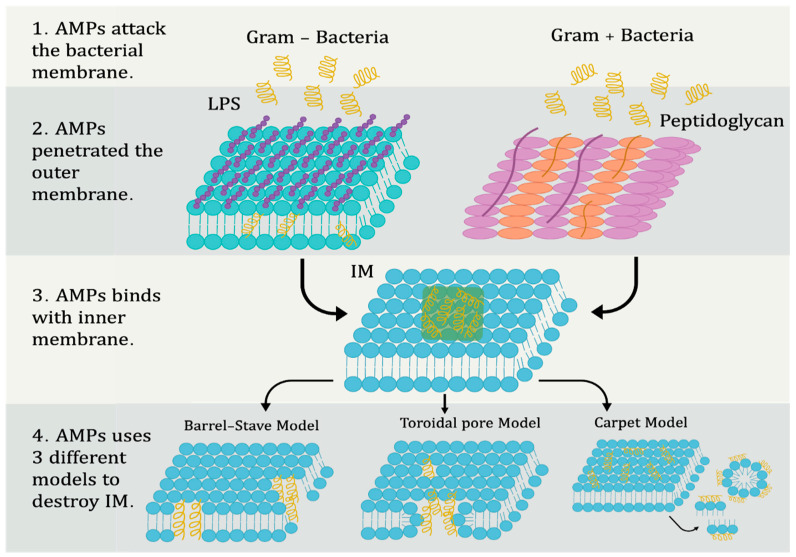
Mechanism of AMPs in membrane targeting.

Antimicrobial peptides (AMPs), otherwise known as host defense peptides (HDPs), have been used by plants and animals as a defense mechanism against infections for thousands of years [88,89,90]. AMPs have broad activity against a variety of pathogens because they bind to the bacterial membrane and fuse with the cytoplasmic membrane to form pores through which the bacteria are killed. In addition, AMPs can emit signals through which they stimulate components of the acquired immune system, such as T cells and dendritic cells [91]. Based on what was reported by the Data Repository of Antimicrobial Peptides (DRAMP), up to 3791 AMPs were discovered from the six kingdoms: the largest number of antimicrobial peptides were found from animals, totalling 2519 AMPs, 824 AMPs from plants, 431 AMPs from bacteria, 7 AMPs from protozoans, 6 AMPs from fungi, and finally 4 AMPs from archaea [89]. In recent years, antimicrobial peptides (AMPs) have attracted significant interest in clinical research to find effective treatment against MDR. This section will focus on the mechanisms of targeting microorganism membranes by AMPs (Figure 4), classifying them according to the amino acid-rich species as shown in Table 2.

### 4.1. Membrane Targeting Mechanism of AMPs

The mechanism of action of AMPs has been studied since their discovery, and has continued until now to fully understand their mechanism of action (MOA). AMPs can directly kill bacteria by interacting with their membrane, and can be divided into membrane-permeable and non-permeable mechanisms of action. Transmembrane-targeting AMPs initiate their reaction from the target bacteria using one of two mechanisms: the non-receptor-mediated or receptor-mediated interaction [92]. For example, nisin is one of the AMPs that are produced by bacteria. Nisin contains two domains: the first domain interacts with lipid II, which is the main peptidoglycan in the cell wall, while the second domain provides a membranous pore-forming ability, which ultimately leads to inhibition of peptidoglycan synthesis and bacterial killing. Indeed, mesentericin has the same mechanism of action as nisin—“receptor-mediated interaction” [93,94].

Most interactions of AMPs with the membranes of the target bacteria begin using a non-receptor-mediated reaction, which depends on electrostatic interaction. This is based on the fact that both Gram-positive and Gram-negative bacteria membranes contain lipopolysaccharides and teichoic acid, which test the membrane with a net negative charge, while AMPs contain cationic residues, which helps in creating an electrostatic force between AMPs and the bacterial membrane, forming a strong mutual bond. It has been found that some types of AMPs, namely lysine and arginine, have a strong affinity for the phosphate group present in the lipid bilayers [92,95]. The most important feature of AMPs is their ability to target the structures that make up the intrinsic difference between the membranes of bacteria and those of multicellular animals. The outer leaflet of the lipid bilayer of the bacterial membrane contains a large proportion of the negatively charged lipids, such as peptidoglycan and cardiolipin. While the outer leaflet of multicellular animal membranes consists of zwitterionic phospholipids such as phosphatidylcholines and neutral components such as cholesterol, the negatively charged lipids are present in the inner leaflet [96,97].

After the initial electrostatic interactions have taken place, the AMPs begin to accumulate on the surface of the bacterial membrane until they reach a certain concentration. When the desired concentration is reached, the AMPs start using different models in order to destroy the bacterial membrane, which leads to the killing of the bacteria [92]. There are different models by which we can describe the mechanism of action of different types of AMPs, and they fall into two main categories: membranous and non-porous pore models. The toroidal pore and barrel-stave models are also divided into two sub-sections [98]. Figure 4 shows the mechanisms of AMPs in bacterial membrane targeting.

#### 4.1.1. Barrel-Stave Models

Initially, the AMPs are oriented parallel to the bacterial membrane, but then their turbulence is modified to be perpendicular to the lipid bilayer [99]. AMPs must be long enough in order to penetrate the bacterial membrane, with the minimum length of peptides being 22 residues (a-helical) and 8 residues (β-sheet) to be able to extend along the lipid bilayer [92,95]. Orientation of AMPs perpendicular to the lateral peptide–peptide interactions leads to the formation of a membrane-protein channel-like structure [92,100]. The formation of pores across the membrane helps to flow out of the cytoplasm, and in cases of severe penetration, AMP stimulation leads to the breakdown of the bacterial membrane, causing bacterial death [98]. A few AMPs use barrel-stave models such as the fungal peptide alamethicin [101], pardaxin [102], and protegrins [103].

#### 4.1.2. Toroidal Pore Models

Toroidal models are also known by another name—wormhole models [98]. AMPs are oriented perpendicular to the lipid bilayer, and unlike barrel-stave models, there are no peptide–peptide interactions [92]. AMPs pull the adipose head into the lipid tail region, resulting in a lipid misalignment of the membrane and altering membrane curvature, resulting in toroidal pores in the membrane [104]. Maganin is the first antimicrobial peptide discovered by working with the toroidal pore model [100]. In addition, melittin stimulates disruption of the lipid bilayer and forms toroidal pores [105].

#### 4.1.3. Carpet Model

In this model, no pores are formed in the membrane, and in this model, AMPs act by disassembling the membrane. Some AMPs are absorbed and are parallel to the bacterial membrane. They begin to accumulate until they reach a threshold concentration, and a “carpet” is formed on the surface of the bacterial membrane; this leads to a loss of membrane integrity by breaking the membrane into pieces by micelles. This mechanism is similar to the way that detergent works [95,102,106]. This model requires a concentration of AMPs, and the influence of β-sheet AMPs is important in this model [98]. AMPs that use a carpet model include cecropin [106], indolicidin [107], LL-37 [108], and aurein 1.2 [109].

### 4.2. Classification of the AMPs Based on Amino Acid-Rich Species

#### 4.2.1. Proline-Rich Peptides

Proline-rich AMPs (PrAMPs) act as antibacterials by permeating the membrane and blocking protein synthesis [110]. Olga Shamova and his associates examined in detail two PrAMPs, OaBac5α and ChBac5, after they were purified from white blood cells of goats and sheep. Both were observed to be broad spectrum antibacterials at low salt concentration, as these peptides remain effective at 100 mM NaCl against *E. coli*, *P. aeruginosa*, *B. subtilis*, and *L. monocytogenes*, but lose their efficacy against *Candida albicans* and *S. aureus* [111]. On the other hand, there are PrAMPs that do not inhibit protein synthesis; for example, Lser-PRP2 and Lser-PRP3 secreted by *Lucilia sericata* larvae act on the wound surface to interact with bacterial chaperone DnaK to prevent protein folding, and they can also make structural changes on the bacterial surface [112].

#### 4.2.2. Tryptophan and Arginine-Rich AMPs

AMPs rich in tryptophan and arginine (Trp- and Arg-rich AMPs) can participate in cationic interactions in the bacterial membrane due to the presence of tryptophan, which has a great effect on the interfacial region of the lipid bilayer, and arginine, which confers cationic charge peptides and bonding properties to hydrogen to enable it to interact with anions circulating in the bacterial membrane, hence its ability to inhibit Gram-negative and Gram-negative bacteria, such as *P. aeruginosa*, *E. coli*, and *S. aureus* [98,113]. These peptides are active with a length of 4 or 5 residues, but if their length is 10 residues, they are at their maximum activity [114].

#### 4.2.3. Histidine-Rich Peptides

Histidine-rich AMPs have therapeutic utility in gene transfer, as well as in cystic fibrosis-associated bacterial infections. Histidine-rich peptides can act as antimicrobials against both Gram-negative bacteria, such as *E. coli* and *Pseudomonas aeruginosa*, Gram-positive bacteria, such as *B. subtilis* and *S. aureus*, and fungi, such as *C. albicans*, at pH 5.5, but their activity is lost at neutral pH. The binding of histidine-rich peptides to *E. coli* and *Candida* was studied under fluorescence microscopy and FACS analysis. In addition, it was noted that these peptides have the ability to hydrolyze the membrane at pH equivalent to 5.5, and that the activity of histidine-rich peptides depends on the pH when interacting with bacteria [115,116].

#### 4.2.4. Glycine-Rich AMPs

Glycine-rich AMPs are considered to have antimicrobial activity against Gram-negative and Gram-positive bacteria, fungi, and occasionally yeasts [117]. Its activity depends on the fragmentation of the microbe due to the profound changes in the structure of the bacteria. Armadillidin H was isolated from blood cells of *Armadillidinium vulgare*; armadillidin H is a glycine-rich peptide with directly potent activity against *Bacillus megaterium*. This was based on what was observed using microscopy on the inability of bacteria to live after armadillidin H reaction caused significant changes in the structure of bacteria [118]. Moreover, glycine-rich cathelicidins contain mature, significantly long peptides, derived from salmonid cathelicidins that potentiate phagocyte-mediated microbial mechanisms [98,119].

**Table 2:** Classification of the AMPs based on amino acid-rich species, biological source, and biological activity.

Amino Acid-Rich Species	Description	Biological Source	Biological Activity	Peptides	Peptide Sequence	Bacteria	MIC (μg/mL)	Animal Model\In Vitro	References
Proline-Rich Peptides	Large group of small and medium in size, heterogeneous, proline-containing peptides that are arranged in peculiar sequences.	Marine sponges (aquatic), microorganisms, and mammals.	Antibacterial and antifungal.	Api88	Gu-ONNRPVYIPRPRPPHPRL-NH_2_	*P. aeruginosa*	32	In vitro	[98,120,121,122,123,124,125]
				Pyrrhocoricin	VDKGSYLPRPTPPRPIYNRN	*K. pneumonia*	4	In vitro	
				Bac7	RRIRPRPPRLPRPRPR	*E. coli*	8	In vitro	
Arginine-Rich AMPs	Composed of small size peptides and has a short cyclic half-life as it is digested by blood proteases.	Frogs (amphibian) and insects	Antibacterial, antiviral, antifungal, antitumor, and immunomodulatory.	Cecropin D	WNPFKELEKVGQRVRDAVISAGPAVATVAQATALAK-NH_2_	*S. aureus*	4.55	In vitro	[126,127,128,129]
				Cecropin B	KWKVFKKIEKMGRNIRNGIVKAGPAIAVLGEAKAL-NH_2_	*H. parasuis*	2	-	
				Cecropin P1	SWLSKTAKKLENSAKKRISEGIAIAIQGGPR-NH_2_	*E. coli*	1000	-	
Histidine-Rich Peptides	Peptides with short sequences, usually H6, and are widely used in the production of recombinant protein as purification tags.	-	Antibacterial, antifungal and antivirus.	HALO	KKALLOHALHOLALLOHLAHOLKKA	*P. aeruginosa*	0.168	In vitro	[130,131,132,133]
				LAH4	(KKALLALALHHLAHLALHLALALKKA)	*E. coli* and *S. aureus*	277.9	In vitro	
Glycine-Rich AMPs	Consists of a series of peptides with a high content of glycine, which has a low profile in animals.	Aquatic and mammals	Antibacterial and antifungal.	Persulcatusin	GFGCPFNQGACHRHCRSIGRRGGYCAGLFKQTCTCYSR-NH_2_	*S. aureus*	0.156–1.25	-	[129,134,135,136]
				Attacins B	QAGALTINSDGTSGAV-VKVPITGNENHKFSALGSVDLT-NQMKL GAATAGLAYDNGNGHGATLT KTHIPGFGDKMTAAGKVNLFHN DNHDFSAKAFATKNMP-NIPQVPNFNTVGAGVDYMFKDKIGASANAAHTDFINRNDYS-LGGKLNLFKTPTTSLDFNAGWKKF DTPFFKSSWEPSTSFSFSKYF	*P. maltophilia*	0.080–0.094	Insect cells	
				Attacins E	DAHGALTLNSDGTSGAVVKVPFAGNDKNIVSAIGSVDLT-DRQKL GAATAGVALDNINGHGLSLTDT HIPGFGDKMTAAGKVNVFHNDNHDITAKAFATRNMPDIANVPN FNTVGGGIDYMFKDKIG TRNMPSIPNVPNFN-TIGGGVDYMYKNKVGASLGMASTPFLDRKDYSAMG WEPNFGFSLSKYF	*E. coli*	0.040–0.046	Insect cells	

### 4.3. Bacterial Resistance to AMPs

The potential of AMPs-resistant bacteria is closely related to the potential of bacterial virulence factors, as both Gram-positive and Gram-negative bacteria have different strategies for AMP resistance [91]. The rate of spread of resistance in a bacterial group under natural conditions can be determined by a complex set of interactions amongst a number of factors, including the rate of mutation and the strength of selection pressure. Recent studies have shown that bacterial mutants resistant to AMPs have the potential to be resistant to a wide range of AMPs with different structures and modes of action [137]. Mechanisms of bacterial resistance to AMPs include proteolysis, biofilm matrix molecules, and modification of the cell surface or membrane to circumvent attraction.

Firstly, proteolysis of AMPs is one of the simplest and most effective methods to develop a defense mechanism for bacteria against AMPs, using extracellular secretory enzymes. For example, *Staphylococcus* secrete several types of proteases such as metalloproteases (ureolysin and SepA) and serine endopeptidases (V8 protease) that linearly degrade human cathelicidin (LL-37( [138,139]. Another type of protease, SpeB, inhibits the action of AMPs such as LL-37 and beta-defensins by fragmenting their host pumps [140,141,142,143]. One of the well-studied groups of proteases found in Gram-negative bacteria is the omptin family. It has been observed that a group of members of this family can cleave AMPs, such as Pla in *Yersinia pestis*, OmpT in *E. coli*, and PgtE in *Salmonella enterica* serotype Typhimurium, which have been shown to cleave AMPs [144,145,146].

Secondly, bacteria with the ability to form biofilms are characterized by up to a hundredfold higher resistance to antibiotics and AMPs compared to planktonic bacteria, which is due to the lower ability of AMPs to penetrate biofilms [91]. Extracellular polymeric substances (EPS) and capsular polysaccharides (CPS) repel or hold AMPs to impede their action, and polysaccharide intercellular adhesin (PIA) is secreted from different groups of bacteria such as *E. coli*, *S. aureus*, *S. epidermidis*, and other *Staphylococcus* species. It works to resist types of AMPs such as cationic HBD-3 and LL-37, as well as anionic dermcidin [147,148]. In addition, IcaB-mediated deacetylation of PIA increases the positive charge of PIA, causing repulsion with CAMPs. This may lead to increased sequestration of dermcidin and formation of a mechanical barrier against AMPs. On the other hand, *Pseudomonas* species are a biomembrane-forming genus whose membranes contain guluronic acid and mannuronic acid that exhibit resistance to AMPs through genetic overexpression, and *P. aeruginosa* may exhibit resistance to LL-37 through EPS, which increases sequestration [149,150,151,152,153].

Thirdly, bacteria can use innate mechanisms to circumvent the attraction of AMPs to their membranes by altering the cell surface or envelope. Important components in the formation of this resistance are teichoic acid (TA) in Gram-positive bacteria and lipopolysaccharide (LPS) in Gram-negative bacteria [91]. Teichoic acids consist of two components, disaccharide anchors and phosphodiester-linked polyglycerol phosphate, which is responsible for the net negative charge of TA. Thus, by adding a positive charge to TA, the attraction between the bacterial surface and AMPs is reduced. This occurs due to the presence of d-alanylation on the free hydroxyl groups of the polysaccharide, which is controlled by the gene products of the dltABCD locus, and which is the reason for the emergence of resistance in *S. aureus* to both HNP-1,2,3, protegrins, magainin II, gallidermin, and nisin [154,155,156]. The d-alanylation of TA is used as a strategy for resistance to AMPs in a variety of bacteria including *Staphylococcus*, *Streptococcus*, and *Bacillus* [91]. Gram-negative peptidoglycans containing the lipid II can be modified to be resistant to AMPs. This is done by replacing the terminal d-alanine, which avoids antibiotics such as vancomycin, and this antibiotic then binds to the d-Ala-d-Ala dipeptide present in the bacterial wall, preventing AMPs from interacting with bacteria [157]. For example, in vancomycin-resistant strains, the terminal d-alanine has been replaced by d-lactate or d-serine, which is resistant hundreds of times [158].

## 5. Conclusions

The bacterial membrane is responsible for conferring resistance while interacting with the host cell and antimicrobials. Processes involving secretion of proteins through various secretion systems and their ability to communicate with their external environment determine cellular density by QS, and is a determining factor on the survivability of the cell. In addition, both systems have a direct effect on the ability of bacteria to form biofilms. Elimination of bacteria through the use of antimicrobials mainly target their membranes, and AMPs, although long known, are only recently gaining more attention for their ability to kill bacteria by making pores in cytoplasmic membranes. Despite the promising role that AMPs can play, some bacteria were still able to develop resistance against AMPs, confounding the MDR issue. AMPs need to be further elucidated for enhanced applications towards the mitigation of AMR worldwide.

## Data Availability

Not applicable.
